# Patterns of Gambling and Other Risky Behaviors among Adolescents. A Latent Class Analysis Study on the 2021/22 Health Behaviors in School Children Survey Data

**DOI:** 10.1007/s10899-026-10479-2

**Published:** 2026-03-04

**Authors:** Giovanni Aresi, Chiara Arienti, Elena Marta, Corrado Celata, Giusi Gelmi, The HBSC Lombardy Committee, Olivia Leoni, Lucia Crottogini, Claudia Lobascio, Lucia Pirrone, Danilo Cereda, Luciana Volta, Simona Chinelli, Giuliana Rocca, Ornella Perego, Paola Ghidini, Lisa A. Impagliazzo, Alida Bonacina, Maria Stefania Bellesi, Lorella Vicari, Laura Rubagotti, Ilenia Fontana, Federica Di Cosimo, Jonathan Molteni, Elisabetta Ferrari, Marina Ghislanzoni, Antonio Cremonesi, Antonella Ferrari, Roberto Manna, Laura Stampini, Angela Sacchi, Annarita Fumarola, Linda Casalini, Maristella Colombo

**Affiliations:** 1 Psychology Department, Università Cattolica del Sacro Cuore, Largo Gemelli 1, 20123 Milano, Italy; 2 CERISVICO Research Centre On Community Development and Organizational Quality of Life, Via Trieste 17, 25121 Brescia, Italy; 3 Azienda Di Tutela Della Salute, Città Metropolitana Di Milano, Corso Italia, 52, 20122 Milan, Italy

**Keywords:** Gambling, Risk behaviors, Behavioral profiles, Adolescence, Latent class analysis, Italy

## Abstract

Research on adolescent gambling often examines this behavior in isolation, overlooking evidence of comorbidity between alcohol/drug use disorders and problem gambling. This study aimed to address this gap by examining patterns of risky behaviors (i.e., substance use and gambling) among a representative sample of 7,795 adolescents aged 15 and 17 living in the Lombardy region of Italy. Latent Class Analysis was used to identify subgroups of individuals characterized by common behavioral patterns. We tested the invariance of gender and age cohort, as well as the relations between class membership, gambling behavior severity, and indicators of health status and life satisfaction. The analysis identified four distinct patterns of risk behaviors across gender and age cohorts. The first pattern is characterized by a low prevalence of risky behaviors. The other three patterns are distinguished by predominant substance use, either a single substance or a combination of multiple substances. Gambling was not observed as a singular behavior within any of these profiles. Rather, it co-occurred with polyconsumption patterns that were associated with higher levels of gambling severity, as well as poorer health and life satisfaction. These findings suggest that a more nuanced understanding of gambling can be achieved by examining it alongside other risk behaviors. During adolescence, a multifaceted health promotion approach that encompasses the prevention continuum and policy responses is necessary to address concurrently shared risk and protective factors of multiple risk behaviors at individual, social, community, and societal levels.

**Supplementary Information:**

The online version contains supplementary material available at https://doi.org/10.1007/s10899-026-10479-2.

The phenomenon of gambling behavior among adolescents has been identified as a significant public health concern (Armitage, 2021). Gambling is generally understood to be a recreational activity that entails the wagering of monetary resources, the outcome of which is not predetermined by the player but is instead determined by chance. Since 2013, with the publication of the DSM-5, pathological gambling has been classified among Substance-Related and Addictive Disorders. According to the DSM-5 definition, gambling disorder is the “*persistent and recurrent problematic gambling behavior leading to clinically significant impairment or distress*” (APA, 2013, p. 585).

According to data from the European School Survey Project on Alcohol and Other Drugs study (ESPAD, 2025), an average of 23% of European students report having engaged in gambling in the past 12 months, either in person or online, through activities such as slot machines, card or dice games, lotteries, or betting on sports or animal races. Italy exhibits the highest rate of gambling among students (45%), followed by Iceland (41%) and Greece (36%). While early exposure to gambling does not invariably result in problematic gambling in adulthood, problems with gambling during adolescence can lead to negative consequences (Edgerton et al., 2015). These include mental health issues, challenges in relationships with family members and friends, and difficulties with school and delinquent behavior (Delfabbro et al., 2016; Kryszajtys et al., 2018; Raisamo et al., 2013). Gambling problems can also cause financial problems (Livazović & Bojčić, 2019).

A notable limitation of extant research on adolescent gambling is its tendency to examine this behavior in isolation, thereby neglecting the evidence on the comorbidity between alcohol/drug use disorders and problem gambling (Gerdner & Håkansson, 2022; Punia et al., 2021). There is evidence that the prevalence of gambling disorder is significantly higher among individuals with substance use disorders than in the general population (Armoon et al., 2025; Zhai et al., 2020). Moreover, as highlighted by Buja et al. (2017), a positive association between gambling and substance use is evident even at levels not classified as risky or problematic. This pattern aligns with established theories of adolescent risk behaviors (e.g., the Problem Behavior Syndrome), suggesting that gambling may serve as a marker of a broader vulnerability to multiple risk-taking behaviors that all share common risk and protective factors at the individual, social, and community levels (Ho, 2017; Jessor, 1992).

Further research is necessary to comprehensively understand the wide range of risky behaviors exhibited by youth and determine whether gambling can be considered a distinct behavior or whether it is better positioned within a more extensive and interconnected network of behaviors. It is noteworthy that adolescents who engage exclusively in gambling activities may exhibit distinct characteristics compared to those who exhibit problem behaviors across a broader spectrum. This is a critical aspect for the advancement of theoretical frameworks that more comprehensively consider gambling as part of a broader constellation of problematic behaviors among adolescents to identify common, cross-cutting risk and protective factors to address in prevention, early identification, and treatment. At the community and societal level, enhancing our comprehension of patterns of adolescent risk behavior can more effectively inform the development of policy strategies aimed at addressing multiple risk behaviors collectively rather than merely addressing them individually.

This study utilized survey data from the 2021/22 Health Behaviors in School Children (HBSC) to examine patterns of risk behaviors (i.e., substance use and gambling) among a representative sample of Italian adolescents aged 15 and 17 years.

## Approaches to Analyzing Patterns of Risk Behaviors

The person-centered approach (e.g., mixture models, latent profile analysis, latent class analysis, cluster analysis, growth mixture analysis) is particularly suitable for identifying common patterns among subgroups in a population (Lanza & Cooper, 2016). In recent years, the application of person-centered statistical techniques has witnessed significant advancements in various fields, including the study of youth (poly)substance (Aresi et al., 2018; Gilreath et al., 2014; Tomczyk et al., 2016). In the domain of gambling studies, numerous investigations have adopted this approach to examine subtypes of adolescent and adult gamblers. However, these studies have predominantly examined this behavior in isolation (Chamberlain et al., 2017; Kong et al., 2014), (Kong et al., 2014; Studer et al., 2016). A recent review of studies on the clustering of health-related behaviors among adolescents (Whitaker et al., 2021) found only three studies that considered the co-occurrence of gambling and substance use using Latent Class Analysis (LCA).Two of these studies are particularly pertinent to the objective of the present study 1^1^. Sullivan et al. (2010) employed a set of twelve risk behaviors (e.g., substance use, driving after drinking, carrying a weapon, and gambling) and identified evidence of four distinct profiles in a sample of high school students in the U.S.A. A group of “High, Diverse Risk Behavior Youth” constituted 22% of the sample and exhibited a high-risk profile across all behaviors, including gambling. In Spain, Martínez-Loredo et al. (2019) employed twelve indicators: ten of which pertained to behaviors (alcohol, tobacco, and cannabis use, in addition to seven distinct gambling activities). The remaining two indicators were measures of the severity of gambling and alcohol problems. A salient finding of the study was the variability of patterns across genders. Among males only, a class characterized by engagement in gambling but not the use of other substances (18.4%) was identified.

The outcomes of LCA are contingent upon the indicators employed; consequently, a direct comparison between the results of these two studies is challenging, and the relationships between gambling and other risk behaviors remain unresolved. A limitation of Martínez-Loredo et al. (2019) is conceptual in regard to the inclusion of severity of gambling and alcohol problems measures in the models, which are better conceptualized as outcomes of risk behavior profiles rather than model indicators. The inclusion of such factors is likely to have exerted an influence on the overall results. Furthermore, both Sullivan et al. (2010) and Martínez-Loredo et al. (2019) did not utilize representative samples, did not examine potential age differences in the taxonomy, and only the latter examined the stability of the model across genders.

## Aim and Hypotheses

The overall goal of this study was to identify behavioral profiles based on a set of risk behaviors that included substance use and gambling. We also examined the consistency of the latent class solution across gender and 15- and 17-year-old age cohorts and how class membership was related to the severity of gambling behavior and indicators of health status (i.e., distal outcome). In accordance with results of a literature review on polysubstance use (Tomczyk et al., 2016), it is anticipated that the identified number of classes will fall within the range of four to seven. Among these classes, one is expected to comprise adolescents who do not exhibit any risk behaviors. Additionally, the presence of multiple latent classes involving (poly)substance use, defined as the consumption of a minimum of two distinct substances, such as alcohol and cannabis, is hypothesized (Hypothesis 1). In light of the findings from prior studies, particularly those by (Martínez-Loredo et al., 2019), it can be posited that there will be variations in the profiles of Italian adolescents across genders (Hypothesis 2). Moreover, it is hypothesized that within the male sample, there will be a group that engages in gambling but not in other substances (Hypothesis 3). It was hypothesized that the identification of profiles would be consistent across age cohorts. However, it was predicted that a greater proportion of older adolescents would engage in risk behaviors compared to 15-year-olds (Hypothesis 4). Finally, in accordance with the extensive extant literature on adolescent polysubstance use (Rodríguez-Cano et al., 2023; Tan et al., 2020; Weller et al., 2020), we hypothesized that latent classes characterized by polysubstance use would exhibit poorer life satisfaction and health outcomes, including severity of gambling (Hypothesis 5).

## Methods

The data were obtained from the Health Behavior in School-aged Children (HBSC) survey of 2021/2022 (Badura et al., 2024). The HBSC is a quantitative, cross-national survey conducted in collaboration with the World Health Organization (WHO). The survey encompasses information gathered from 11-, 13- and 15-year-old boys and girls regarding health-related behaviors during adolescence (Inchley et al., 2023). In Italy, the Lombardy region HBSC workgroup collected data on the additional cohort of 17-year-old students in accordance with international guidelines (Supplemental A).

A total of 17,853 adolescents residing in the Lombardy region participated in the survey (response rate of 96.5%). Of these, 4,272 were 15-year-olds, and 4,651 were 17-year-olds. In the present study, data from the 11- and 13-year-old age groups were excluded due to the absence of available data concerning gambling and other health behaviors in these age cohorts. Following the exclusion of 1,128 cases with missing values on the variables utilized for this study, the analytical sample comprised 7,795 individuals^2^. The prevalence of missing values ranged from 1.5% (N = 141) for tobacco product use to 4.7% (N = 421) for alcohol use. Table 1 presents a comprehensive report on the final sample characteristics.

**Table 1:** Participants’ sociodemographic characteristics

Characteristic	Number (%)
Gender	
Woman	3,754 (48.2)
Cohort	
15 years old	3,614 (46.4)
17 years old	4,181 (53.6)
Both parents born in Italy	6,367 (81.7)
School type	
High school	3,410 (43.7)
Technical school	3,096 (39.7)
Vocational school	733 (9.4)
Other school	435 (5.6)
Missing	121 (1.6)

*N* = 7,795

### Measures

#### Latent Class Indicator Variables

The LCA model was developed with six dichotomic indicators of risk behaviors. These indicators were defined as follows: (1) Any tobacco products (i.e., cigarette and/or heated tobacco cigarette and/or electronic cigarette consumption during the last 30 days); (2) alcohol consumption during the last 30 days; (3) drunkenness during the last 30 days; (4) cannabis consumption during the last 30 days; (5) any other drug (e.g., MDMA) consumption during the last 30 days; and (6) any gambling (i.e., betting or gambling money) during the last 12 months.

#### Distal Outcomes

The severity of problematic gambling behavior was assessed using the *South Oaks Gambling Screen* – Revised for Adolescents (SOGS-RA) (Chiesi et al., 2013). Participants responded to 12 items that assessed the negative consequences associated with gambling behavior over the course of the past year, utilizing a binary scale (No = 0; Yes = 1).

A single item was utilized to assess self-rated health (“Would you say your health is …?”) (Kaplan & Camacho, 1983). Participants responded on a 4-level Likert scale (1 = Excellent; 4 = Poor). The responses were reversed so that higher scores would represent greater health. The objective of this instrument is to assess the subject's comprehensive perception of their health status. The relationship between the phenomenon under consideration and lifestyle has been demonstrated in a number of studies (Braverman et al., 2016; Marques et al., 2019).

The Cantril Ladder was used to measure individuals’ global assessment of their lives (Cantril, 1965). This instrument has been thoroughly validated through the demonstration of its test–retest and construct validity in adolescent samples (see, for example, Mazur et al., 2018). Participants are presented with a depiction of a ladder with ten rungs and are given the following instructions: “The top of the ladder ‘10’ is the best possible life for you and the bottom ‘0’ is the worst possible life for you. In general, where on the ladder do you feel you stand at the moment? Tick the box next to the number that best describes where you stand”.

### Data Analyses

In accordance with the established recommendations (Lanza et al., 2011), a series of LCA statistical models were estimated in the overall sample to identify subgroups of individuals characterized by common patterns of risk behaviors. This was followed by an examination of measurement invariance across gender and age cohorts (15- and 17-year-olds). A detailed exposition of the statistical model fit indices and conceptual standards employed for the comparison of the disparate profile solutions can be found in Sorgente et al. (2019) and Supplemental B. In the final set of analyses, the mean of the three distal outcomes for each of the latent classes by gender and age group was estimated. The analyses were executed in Mplus 7 (Muthén & Muthén, 1998–2015) employing the robust maximum-likelihood estimator.

## Results

Table 2 presents the proportion of participants who reported each risk behavior by gender and age cohort, as well as for the entire sample. A comparison of the prevalence of substance use between genders reveals that men were more inclined to report gambling, cannabis use, and other substance use, while women were more inclined to report tobacco product use. In the study, adolescents aged 17 years old exhibited a higher propensity to report any behavior, with the exception of substance use other than tobacco, alcohol, and cannabis.

**Table 2:** Proportion of respondents reporting risk behaviors, by gender and age cohort

Risk Behavior	Overall (*N* = 7,795)	Gender			Age		
		Women (*N* = 3,754)	Men (*N* = 4,041)	χ2 test	15-years-old (*N* = 3,614)	17-years-old (*N* = 4,181)	χ2 test
Any tobacco	38.1	41.6	34.9	36.373***	32.3	43.2	97.692***
Any alcohol	66.7	67.2	66.3	0.730	55.8	76.2	364.059***
Any drunkenness	21.8	20.6	21.8	1.782	15.7	26.0	122.734***
Any cannabis	18.8	15.8	20.4	28.572***	13.4	22.4	105.468***
Any other substance	6.9	6.2	7.5	5.446*	6.5	7.2	1.449
Any gambling	22.3	10.3	33.5	604.771***	20.0	24.4	21.805***

All indicators refer to the last 30 days, with the exception of any gambling, which refers to the previous year. Values indicate the percentage of respondents who reported the behavior. N = sample size. * *p* < 0.05, ** *p* < 0.01, *** *p* < 0.001

### Identification of Latent Classes of Risk Behaviors

A comparative analysis was conducted among models comprising two to seven latent classes. However, the seven-class model, as well as the six-class model for both age cohorts, did not converge and were not reported. The inspection of fit indices provided evidence to support the four-class solution in all samples (Table 3). In all cases, the selected models exhibit acceptable entropy values. In the women sample, the three-class solution was also considered for parsimony and because the approximate correct model probability (cmP) value suggested that this model could be considered valid. However, the entropy value was found to be below the acceptable threshold. Furthermore, the selection of a model with a different number of classes would have rendered the comparison of models across genders unfeasible. In light of the aforementioned factors, the decision was made to implement the four-class model.

**Table 3:** Model fit statistics for LCA models with two to six latent classes

Model	*-LL*	SCF	χ2 *LRT*	Stdres %	LMR- LRT	BLRT	CAIC	ssBIC	BF	cmP	SSS	Entropy
Overall sample												
2-class	-21,131.472	1.08	1,025.874***	55.00	***	***	42,288.944	42,338.13	0.00	0.0000	3,266	0.742
3-class	-20,827.672	1.02	1,025.874***	12.70	***	***	41,695.343	41,771.01	0.00	0.0000	1,294	0.700
4-class	**-20,762.663**	**1.03**	114.174***	**5.56**	***	***	**41,579.325**	**41,681.48**	**4.23**	**0.8088**	**117**	**0.749**
5-class	-20,732.741	1.03	52.223***	0.00	***	***	41,533.482	41,662.12	168593322.98	0.1912	141	0.699
6-class	-20,720.320	1.02	27.990*	0.00	n.s	***	41,522.639	41,677.76	-	0.0000	62	0.745
Women												
2-class	-9353.781	1.04	422.226***	23.40	***	***	18,733.563	18,773.253	0.00	0.0000	1,608	0.730
3-class	-9230.439	1.02	147.182***	4.26	***	***	18,500.879	18,561.940	59.89	1.0000	601	0.670
4-class	**-9205.725**	**1.02**	73.412 ***	**0.00**	***	***	**18,465.449**	**18,547.882**	**39173066.91**	**0.0167**	**40**	**0.724**
5-class	-9194.401	1.03	43.397*	0.00	***	*	18,456.802	18,560.606	6774964744.95	0.0000	50	0.832
6-class	-9188.231	1.02	19.701***	0.00	n.s	n.s	18,458.461	18,583.637	N/a	0.0000	50	0.772
Men												
2-class	-11364.039	1.13	610.457***	31.37	***	***	22,754.079	22,794.726	0.00	0.0000	1,629	0.759
3-class	-11180.344	1.01	195.457***	5.88	***	***	22,400.687	22,463.221	0.00	0.0000	726	0.719
4-class	**-11130.544**	**1.04**	112.821***	0**.**00	***	***	**22,315.087**	**22,399.508**	**194366.34**	**1.0000**	**334**	**0.760**
5-class	-11113.656	1.01	73.338***	0.00	***	***	22,295.312	22,401.620	16601440.06	0.0000	382	0.681
6-class	-11101.216	1.00	25.454	0.00	*	***	22,284.432	22,412.627	N/a	0.0000	22	0.852
15-year-old cohort												
2-class	-9126.438	1.03	404.893***	28.85	***	***	18,278.876	18,318.072	0.00	0.0000	1,213	0.774
3-class	-9020.175	1.03	153.720***	3.85	***	***	18,080.349	18,140.651	0.62	0.3834	491	0.733
4-class	**-8991.026**	**1.08**	68.727***	**0.00**	***	***	**18,036.051**	**18,117.458**	**661655.30**	**0.6166**	**72**	**0.769**
5-class	-8975.754	1.03	36.583	0.00	*	***	18,019.508	18,122.021	0.00	0.0000	72	0.745
17-year-old cohort												
2-class	-11772.432	1.10	545.907***	31.25	***	***	23,570.863	23,611.953	0.00	0.0000	1,462	0.711
3-class	-11588.178	1.03	154.321***	6.25	***	***	23,216.356	23,279.570	0.00	0.0000	831	0.678
4-class	**-11548.968**	**1.03**	70.149***	**2.08**	***	***	**23,151.935**	**23,237.275**	**33.46**	**1.0000**	**362**	**0.735**
5-class	-11534.416	1.05	34.716	0.00	*	***	23,136.831	23,244.296	0.00	0.0299	62	0.679

*LL* log likelihood; *SCF* scaling correction factor of the robust maximum likelihood estimator; χ2 *LRT* likelihood ratio chi square goodness-of-fit; *Stdres* standardized residuals; *LMR-LRT* Lo–Mendell–Rubin likelihood ratio test; *BLRT* bootstrapped likelihood ratio test; *CAIC* Consistent Akaike information criterion; *ssBIC* sample-size adjusted Bayesian information criterion; *BF* Bayesian factor; *cmP* approximate correct model probability. *SSS* smaller class numerosity. * *p* < 0.05, ** *p* < 0.01, *** *p* < 0.001, n.s. = *p* > 0.05; Selected model in bold

### Assessing Gender and Age Cohort Invariance of Item Response Probabilities

The fully invariant model (in which all parameters were constant across gender and age groups) was statistically different from the baseline model (Table 4). Consequently, the assumption of full measurement invariance could not be made. In an effort to obtain a partially invariant model statistically equal to the baseline, we attempted to allow for variability across gender and age groups. However, despite the implementation of appropriate starting values, the software was unable to accurately accommodate comparable classes, owing to the marked heterogeneity observed among some of them (e.g., the tobacco class and the multiple risk behaviors, the absence of drunkenness classes in both male and female samples). This rendered the allowance of free parameters an impractical solution. This finding lends further credence to the notion that there are pronounced differences in the model between genders and age cohorts.

**Table 4:** Chi-square difference tests by gender and age cohort based on log likelihood values

	-LL	SCF	*d*	Δ	*df*	*p-value*
Gender invariance						
Baseline model	-25,734.200	1.03	55			
Fully invariant model	-25,936.969	1.20	31	506.843	24	< 0.001
Age cohort invariance						
Baseline model	-25,922.440	1.04	55			
Fully invariant model	-25,958.390	1.02	31	68.091	24	< 0.001

*LL* model log likelihood; *SCF* scaling correction factor of the robust maximum likelihood estimator; *d* number of free parameters; Δ difference test value; *df* degree of freedom of the difference test

### Interpretation of the Classes by Gender and Age Cohort Groups

**Fig. 1:**
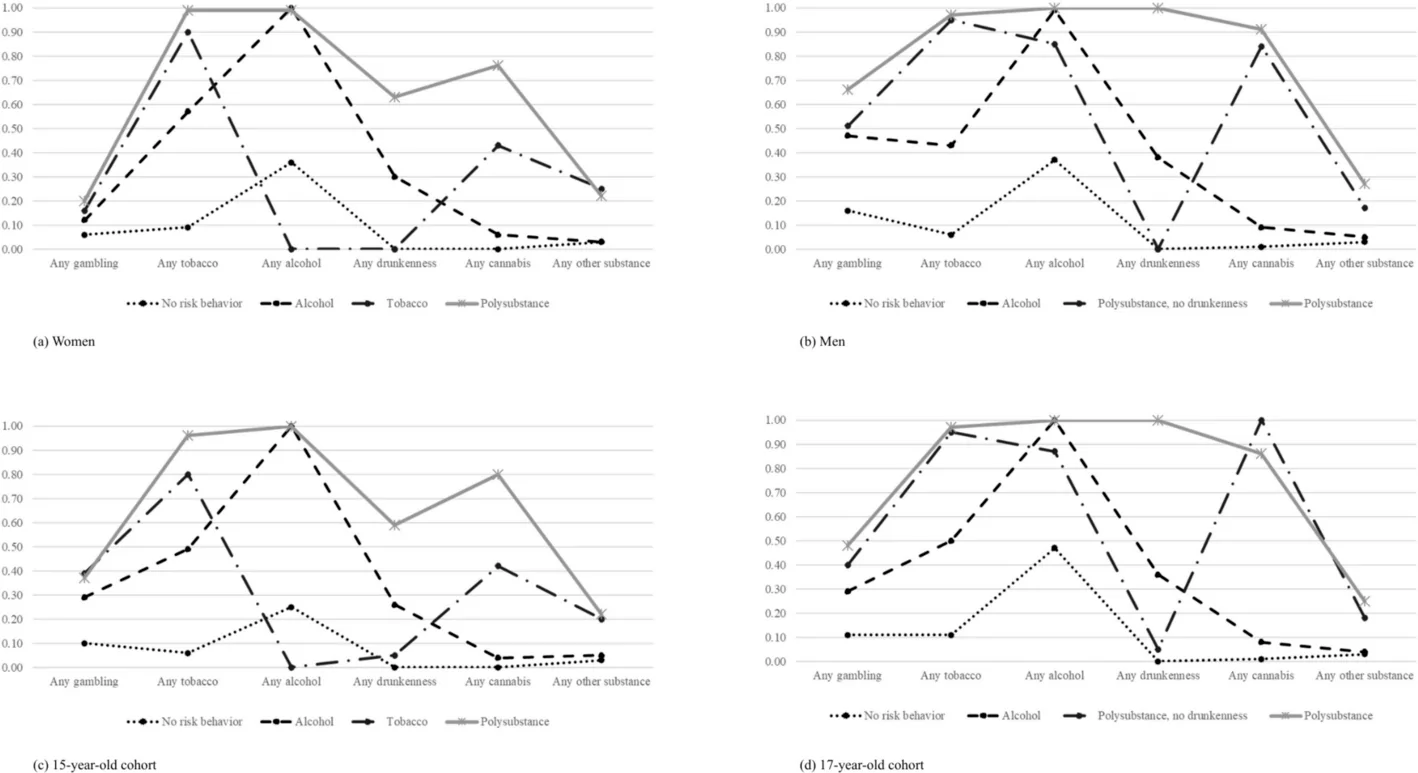

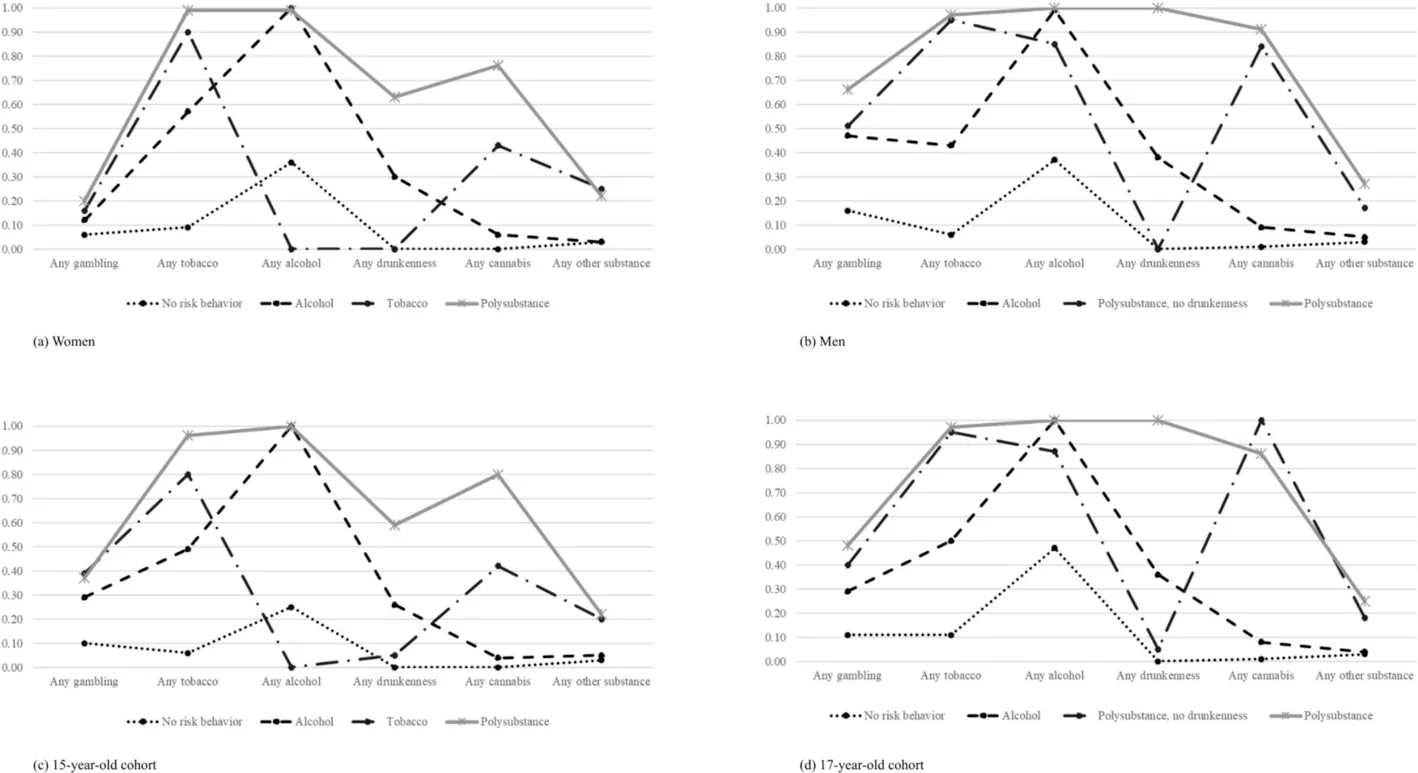
The four-class measurement model by gender and age cohort

Tables 5 and 6 present the results of the selected four-class model in the subsamples by gender and age cohorts, whereas Supplemental C reports results in the overall sample. Figure 1 provides a visual representation of the results by gender and age cohort. The subsequent numerical values appended to each subgroup heading (item response probabilities) denote the probability that the participants in each latent class reported exhibiting a specific behavior (1 = 100%). Class interpretation is not reported for the overall sample due to the fact that examination of item response probabilities separately by gender and age cohort revealed important differences in the meaning of the classes. Among both women and men, the largest class exhibited an absence of risk behavior, as indicated by the low probability of alcohol consumption ("*No risk behavior*"). Among men and women, about 30–33% of the sample, engaged primarily in alcohol use (“*Alcohol*”). The class in question was characterized by a relatively high probability of concomitant use of tobacco products, particularly among women, and, for men only, gambling. Among the male subjects, two distinct classes were identified, comprising approximately 20% of the total sample. These classes exhibited a propensity for engaging in multiple risk behaviors, including the use of tobacco, alcohol, and cannabis, along with gambling to a relatively high degree. The distinction between the two groups was based on whether they reported experiencing drunkenness (“*Polysubstance*”) or not (“*Polysubstance, no drunkenness*”). However, a divergent pattern was identified in the female population: A relatively sizable class (17%) was identified, exhibiting a pattern of "polysubstance" use, excluding gambling. Concurrently, a smaller class (1.8%) was identified, characterized exclusively by tobacco use.

**Table 5:** Item response probabilities for the four-class LCA model, by gender

Latent Class				
	Women			
	No risk behavior	Alcohol	Tobacco	Polysubstance
Any gambling	0.06	0.12	0.16	0.20
Any tobacco	0.09	**0.57**	**0.90**	**0.99**
Any alcohol	0.36	**1.00**	0.00	**0.99**
Any drunkenness	0.00	0.30	0.00	**0.63**
Any cannabis	0.00	0.06	0.43	**0.76**
Any other substance	0.03	0.03	0.25	0.22
**Estimated Prevalence**	48.3%	33.0%	1.8%	16.9%
	Means of distal outcomes			
SOGS-RA	0.297	0.308	0.737	0.422
Self-rated health	2.812	2.838	2.643	2.694
Life satisfaction	6.299	6.278	5.563	5.734
	Men			
	No risk behavior	Alcohol	Polysubstance, no drunkenness	Polysubstance
Any gambling	0.16	0.47	**0.51**	**0.66**
Any tobacco	0.06	0.43	**0.95**	**0.97**
Any alcohol	0.37	**0.99**	**0.85**	**1.00**
Any drunkenness	0.00	0.38	0.00	**1.00**
Any cannabis	0.01	0.09	**0.84**	**0.91**
Any other substance	0.03	0.05	0.17	0.27
**Estimated Prevalence**	50.6%	29.7%	9.1%	10.5%
	Means of distal outcomes			
SOGS-RA	0.463	0.581	1.017	1.008
Self-rated health	3.169	3.155	3.083	3.021
Life satisfaction	6.788	6.83	6.434	6.408

**Table 6:** Item response probabilities for the four-class LCA model, by age cohort

Latent Class				
	15-year-old cohort			
	No risk behavior	Alcohol	Tobacco	Polysubstance
Any gambling	0.10	0.29	0.39	0.37
Any tobacco	0.06	0.49	**0.80**	**0.96**
Any alcohol	0.25	**1.00**	0.00	**1.00**
Any drunkenness	0.00	0.26	0.05	**0.59**
Any cannabis	0.00	0.04	0.42	**0.80**
Any other substance	0.03	0.05	0.20	0.22
Estimated Prevalence	55.6%	28.0%	2.6%	13.9%
	Means of distal outcomes			
SOGS-RA	0.378	0.448	0.912	0.874
Self-rated health	3.029	2.993	2.929	2.885
Life satisfaction	6.597	6.53	6.001	6.139
	17-year-old cohort			
	No risk behavior	Alcohol	Polysubstance, no drunkenness	Polysubstance
Any gambling	0.11	0.29	0.40	0.48
Any tobacco	0.11	**0.50**	**0.95**	**0.97**
Any alcohol	0.47	**1.00**	**0.87**	**1.00**
Any drunkenness	0.00	0.36	0.05	**1.00**
Any cannabis	0.01	0.08	**1.00**	**0.86**
Any other substance	0.03	0.04	0.18	0.25
Estimated Prevalence	42.7%	36.7%	8.2%	12.5%
	Means of distal outcomes			
SOGS-RA	0.510	0.452	0.984	0.799
Self-rated health	2.975	2.981	2.913	2.887
Life satisfaction	6.564	6.489	6.08	6.119

With respect to the two age cohorts under consideration, the prevalence of "*No risk behavior*" was found to be lower in the older cohort (42.7%) compared to the younger cohort (55.6%). Among 15-year-olds, a relatively substantial proportion (28%) exhibited alcohol and, to a lesser extent, tobacco product use ("*Alcohol*"). This class was also present in the 17-year-old sample but exhibited a higher prevalence rate of 36.7%. A small "*Tobacco*" class (2.6%) comprising individuals who consumed exclusively tobacco products and, to a lesser extent, cannabis, was identified in the younger cohort but not in the older one. Among the 15-year-old sample, approximately 14% of the subjects exhibited polysubstance use. Finally, among 17-year-olds, two classes were identified that exhibited a high degree of risk behaviors, including tobacco, alcohol, and cannabis use, as well as frequent gambling. The two classes under consideration here are analogous to those found in the male subgroup. Collectively, these two classes comprise approximately 20% of the sample.

### Associations between Latent Classes and Distal Outcomes

The estimated means of the three distal outcome variables for each of the four latent classes are displayed in Tables 5 and 6. An examination of statistically significant pairwise mean differences across the classes by gender and age groups reveals underlying patterns (Table 7). Firstly, the "*No risk behavior*" and the "*Alcohol*" classes exhibited no significant differences in terms of gambling severity, self-rated health, and life satisfaction outcomes. This finding suggests that these groups may be characterized as relatively low risk, given their tendency to engage in behaviors that demonstrate minimal impact on their overall health and life satisfaction. In contrast, individuals classified as "*Polysubstance*" exhibited the most pronounced gambling severity, with the exception of women. These individuals also reported the lowest ratings of self-rated health and life satisfaction. A notable finding was the similarity in outcomes observed among these groups and those classified as "*Polysubstance, no drunkenness*" among men and 17-year-olds. This finding suggests that abstinence from alcohol does not appear to offer a protective effect for this group, as alternative behaviors (e.g., tobacco and cannabis use) may still impose a significant burden on their health. The "*Tobacco*" classes, which included women and 15-year-olds, exhibited outcomes that fell between the low-risk ("*No risk behavior*" and "*Alcohol*") and high-risk classes ("*Polysubstance*"). For instance, women in the "*Tobacco*" class exhibited a comparable gambling severity and health perception to those in the low-risk classes, yet they reported a lower level of life satisfaction. A similar trend was observed among 15-year-olds.

**Table 7:** Results of pairwise comparisons of mean scores of distal outcomes by class

	Wald test χ^2^					
	1 vs. 2	1 vs. 3	1 vs. 4	2 vs. 3	2 vs. 4	3 vs. 4
	Women					
SOGS-RA	0.005	0.438	0.830	0.428	0.573	0.223
Self-rated health	0.799	3.468	16.716***	2.647	8.849**	0.231
Life satisfaction	0.076	6.277*	37.203***	5.883*	27.582***	0.327
	Men					
SOGS-RA	2.025	15.341***	20.831***	9.587**	13.118***	0.004
Self-rated health	0.212	4.233*	14.225***	2.296	8.639**	1.388
Life satisfaction	0.364	10.415**	14.476***	10.937**	15.205***	0.036
	15-year-old cohort					
SOGS-RA	0.575	2.628	13.635***	2.100	9.182**	0.012
Self-rated health	1.305	1.155	14.048***	0.466	5.677*	0.206
Life satisfaction	0.704	7.229**	21.028***	5.639*	11.476**	0.356
	17-year-old cohort					
SOGS-RA	0.262	9.969**	7.727**	8.963**	6.275*	1.139
Self-rated health	0.041	2.295	5.874*	1.782	4.618*	0.216
Life satisfaction	1.094	11.945**	15.291***	15.902***	20.097***	0.077

The variables are categorized by gender and age cohort

1 = No risk behavior, 2 = Alcohol, 3 = Tobacco (women and 15-year-olds), Polysubstance, no drunkenness (men and 17-year-olds), 4 = Polysubstance; * *p* < 0.05, ** *p* < 0.01, *** *p* < 0.001

## Discussion

This study makes a significant contribution to our understanding of adolescent risk behaviors with regard to substance use and gambling. It does so by examining data from a large, representative sample of adolescents aged 15 and 17 years residing in the Lombardy region of Italy. In contrast to the predominant tendency in the field to examine gambling in isolation, our study adopted a more comprehensive approach by considering gambling as part of a broader constellation of behaviors (Jessor, 1992).

The results indicated the presence of four distinct gender and age cohort variant classes. Our first hypothesis was confirmed, as evidenced by the identification of a behavioral profile characterized by the absence of risk behaviors among adolescents. This behavioral profile constituted a proportion of participants ranging from 42.7% in the 17-year-old group to 55.6% in the younger group of 15-year-olds. This proportion is analogous to that reported by Martínez-Loredo et al. (2019) among Spanish adolescents. The remaining behavioral profiles manifest patterns involving (poly)substance use, defined as the consumption of two or more distinct substances.

We found that the classes were gender variant (Hypothesis 2); however, we did not find evidence of a group characterized by engagement in gambling but not in other substances (Hypothesis 3). This outcome was somewhat unexpected since a prior study in Spain had identified a relatively large class (18.4%) of adolescent males who exhibited engagement in gambling but not in other substances (Martínez-Loredo et al., 2019). Notwithstanding cultural and societal variations between Italy and Spain, the existence of their geographical and cultural contiguity suggests the probable explanation is that of statistical or sample artifact (Aresi & Bloomfield, 2021; Aresi et al., 2022, 2023a, 2023b). Specifically, Martínez-Loredo et al. (2019) developed a model that incorporated a total of twelve indicators, eight of which pertained to distinct gambling activities and severity. This approach predominantly accentuated gambling in the model favoring the presence of a class of gamblers.

The analyses indicated that the classes exhibited variation by age cohorts (Hypothesis 4). The result that older adolescents were more likely to consume substances aligns with the substantial body of literature indicating that adolescence, specifically between the ages of 15 and 17, constitutes a critical period for the onset of substance use behaviors (see, for example, Brennan et al., 2025). The novelty of this study is evidenced by its findings that the combination of substances used also varies. The proportion of polysubstance users results increased at 17 years. However, it is essential to acknowledge that the repeated cross-sectional approach of this study precludes the possibility of entirely excluding the potential for these results to be indicative of cohort differences rather than developmental trends.

Finally, latent classes characterized by polysubstance use exhibited poorer health outcomes, including more severe gambling behavior (Hypothesis 5). This finding aligns with the results of previous studies and provides further evidence that validates the classification developed in this study (Rodríguez-Cano et al., 2023; Tan et al., 2020; Weller et al., 2020). The present study also demonstrates that the association between the severity of gambling and substance use is particularly pronounced among high-risk polysubstance users (Buja et al., 2017).

### Strengths, Limitations and Future Research Directions

The study's primary strengths lie in the size and representativeness of the sample, as well as the implementation of a person-centered approach to identify a categorical latent structure underlying patterns of risk behaviors. LCA is a robust statistical method that boasts numerous advantages (Lanza & Cooper, 2016); however, it is essential to recognize its limitations (i.e., class misassignment and subjectivity in model selection) (Weller et al., 2020). In order to address the aforementioned limitations, the present study followed established recommendations and utilized multiple fit statistics. Subsequent studies should examine the stability of the classification system identified and the extent to which it is grounded in real-world behavior.

The utilization of single items to assess the distal outcomes of LCA models may not be optimal, as these measures may lack the depth and complexity inherent in multi-item scales. Nonetheless, research has demonstrated the capacity of such measures to offer valid insights into constructs such as general well-being and health satisfaction (DeSalvo et al., 2006). These measures can function as reasonable substitutes for more extensive instruments. The present study is subject to further limitations related to the bias introduced by the self-administered nature of the questionnaire and the measures used that were constrained to those that were available in the HBSC survey. For instance, the HBSC survey assesses gambling behavior over the past year, whereas substance use and other risk behaviors are measured over the past month. This discrepancy in the timeframes of the items may have influenced our results, though the specific effects cannot be predicted.

It is essential that future research endeavors focus on further corroborating the behavioral profiles identified in the present study. Such efforts should extend to the collection of additional samples, encompassing diverse geographical regions within Italy and other nations. The samples should be drawn from a range of social, cultural, and policy contexts. This methodological approach is expected to facilitate a comprehensive and nuanced examination of the observed phenomena. Additionally, it would be advantageous to ascertain the stability of these behavioral profiles as young individuals transition into young adulthood, a period during which risky behaviors tend to peak, and subsequently into full adulthood (Aresi & Pedersen, 2016; Arnett, 2005). Achieving this objective necessitates the implementation of longitudinal research methodologies.

### Practice Implications

The primary practical implication that can be drawn from this study is that youth gambling appears to be one component of a broader syndrome of maladjustment that, for a significant proportion of adolescents, manifests in conjunction with substance use. Consequently, while targeted interventions are necessary for the high-risk group of adolescents (15–20%) who consume multiple substances and gamble (Hsiung et al., 2022), for the majority of adolescents and prior to the crystallization of problematic or addictive behaviors (Merrin & and Leadbeater, 2018), a multifaceted health promotion approach is more likely promote the well-being of young individuals and facilitate their successful transition to adulthood. Interventions targeting gambling in isolation have demonstrated their value (Davis et al., 2025). However, the limitations of a single-intervention approach to achieving changes in complex social systems, such as schools and communities, are increasingly recognized (Moore et al., 2019). Integrated interventions that authentically engage community partners and build the capacity of community settings to address shared etiological factors can more efficiently, cost-effectively, and sustainably prevent a range of problematic behaviors (Botvin & Griffin, 2014b; Cassetti et al., 2020; Tengland, 2016). Accordingly, from a socio-ecological framework that conceptualizes behaviors by taking into account shared risk factors and protective factors at the individual, social, community, and societal levels (Bronfenbrenner & Morris, 2007; Stokols, 1996), programs encompassing the entire range of the prevention continuum aimed at fostering safe and nurturing family, school, and community environments are of paramount importance in mitigating stress and facilitating the optimal development of young individuals (Aresi et al., 2023a, 2023b; Barr, 2018; Langford et al., 2015; Mihić et al., 2022; Paradisi et al., 2024; Twum-Antwi et al., 2020).

The consideration of policy is also paramount for the long-term viability of health promotion endeavors. However, policy has historically focused on addressing individual risk domains, neglecting the co-occurrence of risk behaviors in youth (Hale & Viner, 2012). The Lombardy Regional Council has established Regional Resolution 6761/22, which stipulates the provision of Local Plans for Health Promotion and the Prevention of Behavioral Risk Factors. These plans are consistent with the World Health Organization's (World Health Organization, 2017) health promotion approach and emphasize the identification and evaluation of more sophisticated methods for problem identification and problem-solving across various health domains by institutions and social organizations. These methods involve collaborative and community-based approaches with adolescents and the adults who interact with them. In the Lombardy region, the Life Skills Training (LST) program constitutes a concrete application of this approach (Botvin & Griffin, 2014a). The LST program underwent a process of cultural adaptation for Italy (Velasco et al., 2015, 2017), and the Lombardy Region has also implemented the program with the objective of preventing gambling behavior. The program has been scientifically validated as an educational initiative aimed at promoting health in school populations through the development of core cognitive, social, and emotional skills. These skills are deemed essential for effective interpersonal relations and for coping with the pressures and stresses of daily life. A salient and distinguishing attribute of this program is the active participation of educators in its implementation and delivery. Intervening through key educational figures aligns with a community-based intervention framework, which seeks to modify students' living and learning contexts in order to create optimal conditions in which the environment is less conducive to the adoption of risk behaviors and instead functions as a protective factor.

## Conclusions

This study has made an original contribution to the research literature by examining patterns of risk behaviors (i.e., substance use and gambling) among a large, representative sample of adolescents. The findings of the present study indicate that a more nuanced understanding of gambling can be achieved by analyzing it in conjunction with other risk behaviors. A multifaceted approach that encompasses the entire range of the prevention continuum, address system-level change at individual, school and community level, and is supported by relevant health promoting policy is necessary to address the underlying social and environmental influences that are common to those behaviors in adolescence.

## Supplementary Information

Below is the link to the electronic supplementary material.

Supplementary file1 (DOCX 53 KB)

## Data Availability

The data supporting the findings of this study were licensed for use in the current study from the HBSC Lombardy Region Coordinator Committee. The data that support the findings of this study are available from the third author, CC, upon reasonable request.

## Footnotes

1 The objective of the study conducted by Hasking et al. (2011) was to identify typologies of delinquency. Gambling was one of 51 indicators incorporated into the models. Consequently, it plays a rather modest role in the identification of the classes and virtually none in the interpretation of their meaning.

2 Removing cases with missing values on indicators included in the model is a prerequisite of LCA.
