# Supplementary material for: Patterns of Gambling and Other Risky Behaviors among Adolescents. A Latent Class Analysis Study on the 2021/22 Health Behaviors in School Children Survey Data
Source: J Gambl Stud. 2026 Mar 4;42(3):905–24. doi: 10.1007/s10899-026-10479-2 (PMC13578095; doi:10.1007/s10899-026-10479-2)
Supplement: Supplementary file 1 [file 10899_2026_10479_MOESM1_ESM.docx]

# Supplemental A.

### 2021/2022 HBSC Study Protocol in Italy and Lombardy Region

The 2021/2022 Italian HBSC study was promoted by the National Health Institution in collaboration with a national academic network, the Ministry of Education and Health, and by the regional government and regional school office. The HBSC international study protocol was followed to define the instruments, participant consent procedures, anonymity, confidentiality, and data collection and processing (Inchley et al., 2023). The HBSC study protocol and questionnaire were formally approved at the national level by the Bioethics Committee of the University of Torino, by the regional school office, and by the regional government committee. In Italy, the survey employs a national sample and utilizes representative samples for each region. In Lombardy, a formal presentation of the survey was disseminated to all schools, and sampled schools were invited to participate. All sampled schools were contacted to promote participation by the coordinators of the survey, the local school offices, and the local health units. The survey was administered during the school day by health professionals who had undergone specialized training. The participants were informed about the study, consented to participate, and were assured that their data would be kept confidential.

**References**

Inchley, J., Currie, D., Samdal, O., Jåstad, A., Cosma, A., & Nic Gabhainn, S. (2023). *Health Behaviour in School-aged Children (HBSC) Study Protocol: background, methodology and mandatory items for the 2021/22 survey*. MRC/CSO Social and Public Health Sciences Unit, University of Glasgow.

# Supplemental B.

### LCA Fit Indexes

The comparison of LCA models is conducted based on statistical standards, including absolute and relative model fit indices, as well as conceptual standard (Nylund, 2007). As measures of absolute model fit, both the likelihood ratio chi-square goodness of fit (χLRT2) and the standardized residuals for each response pattern can be consulted. Specifically, the best model should be the one with the fewest number of classes that did not reject the χLRT2 null hypothesis (p-value > .05) and where the number of standardized residuals with large values (i.e., > |3|) does not exceed 5% (Masyn, 2013). In order to assess the relative model fit, it is necessary to utilize both statistical tests and descriptive measures. The statistical tests that can be adopted are the adjusted Lo-Mendell-Rubin likelihood ratio test (adjusted LMR-LRT) (Lo et al., 2001) and the parametric bootstrapped likelihood ratio test (BLRT) (McLachlan & Peel, 2000). A statistically significant p-value indicates that the k-class model (where k is the number of classes) fits the data significantly better than a model with one less class. To assess the relative model fit, four distinct measures were employed. Specifically, two information criteria were employed: the Consistent Akaike Information Criterion (CAIC) and the sample-size adjusted Bayesian Information Criterion (ssBIC), where smaller values are indicative of a superior fit. In addition, the approximate Bayes factor (BF) and the approximate correct model probability (cmP), both of which were introduced by Nagin (1999), were utilized. The BF is a statistical method that compares two models at a time (k and k+1 models). The most parsimonious k-class model with BF > 3 is identified as the best model. In contrast, the cmP is a statistical method that compares all models under consideration. In this case, any model with a cmP greater than .10 could be considered a candidate model. Subsequent to the selection of the optimal solution(s), an examination of entropy values was conducted as a means to summarize the classification accuracy. However, these values were not employed to ascertain the optimal number of profiles (Lubke & Muthén, 2007). As demonstrated in the research by Fonseca and Cardoso (2007), entropy values that approximate 1.00 are indicative of superior case classification, with the 0.70 cut-off frequently employed in such analyses.

**References**

Fonseca, J. R., & Cardoso, M. G. (2007). Mixture-model cluster analysis using information theoretical criteria. Intelligent Data Analysis, 11(2), 155-173. https://doi.org/10.3233/ida-2007-11204

Lo, Y., Mendell, N. R., & Rubin, D. B. (2001). Testing the number of components in a normal mixture. Biometrika, 88(3), 767-778. https://doi.org/10.1093/biomet/88.3.767

Lubke, G., & Muthén, B. O. (2007). Performance of Factor Mixture Models as a Function of Model Size, Covariate Effects, and Class-Specific Parameters. Structural Equation Modeling: A Multidisciplinary Journal, 14(1), 26-47. https://doi.org/10.1080/10705510709336735

Masyn, K. E. (2013). Latent class analysis and finite mixture modeling. In T. D. Little (Ed.), The Oxford Handbook of Quantitative Methods: Vol. 2. Statistical Analysis (pp. 551-611). Oxford University Press.

McLachlan, G., & Peel, D. (2000). Finite mixture models. Wiley.

Nagin, D. S. (1999). Analyzing developmental trajectories: A semiparametric, group-based approach. Psychological Methods, 4(2), 139-157. https://doi.org/10.1037/1082-989X.4.2.139

Nylund, K. L. (2007). Latent Transition Analysis: Modeling Extensions and an Application to Peer Victimization (Doctoral dissertation) https://www.statmodel.com/download/nylunddis.pdf

# Supplemental C.

**Table S1.**

### Item Response Probabilities for the Four-class LCA Model for the Full Sample (N = 7,795).

| **Latent Class** | | | | |  |
| --- | --- | --- | --- | --- | --- |
|  |  | | | |  |
|  | No risk behavior | Tobacco, alcohol | Tobacco, cannabis | Polysubstance | |
| Any gambling | 0.11 | 0.29 | 0.36 | 0.43 | |
| Any tobacco | 0.08 | **0.52** | **0.81** | **0.98** | |
| Any alcohol | 0.36 | **1.00** | 0.00 | **1.00** | |
| Any drunkenness | 0.00 | 0.33 | 0.04 | **0.62** | |
| Any cannabis | 0.00 | 0.09 | **0.56** | **0.88** | |
| Any other substance | 0.03 | 0.05 | 0.24 | 0.23 | |
| **Estimated Prevalence** | 53.0% | 30.4% | 1.5% | 15.5% | |

In the aggregate sample, the majority group (53.0%) exhibited minimal propensity for engaging in risk behaviors, including gambling, with the lowest observed probability being 0.10. This group was designated "*No risk behavior*". The remaining three classes exhibited a tendency towards polysubstance use. The second largest group (30.4%) exhibited two predominant behaviors: tobacco and alcohol use, albeit with a relatively low probability of intoxication. The group in question was designated "*Tobacco, alcohol*". A third, diminutive class (1.8%) was distinguished by the utilization of tobacco and cannabis, excluding alcohol. The aforementioned group was designated "*Tobacco, cannabis*". Approximately 15% of the sample was classified as "*Polysubstance*." The distinguishing characteristic of this class is the elevated probability of alcohol use, heavy drinking, tobacco consumption, and cannabis consumption. This group was characterized by the highest probabilities of engaging in gambling (0.43). However, no group exhibited a prevalence of gambling behaviors. Consequently, the term "gambling" was excluded from all class labels.
